# Opportunities to Target T Cell Trafficking in Pediatric Inflammatory Bowel Disease

**DOI:** 10.3389/fped.2021.640497

**Published:** 2021-03-18

**Authors:** Eirini Giannoudaki, Siobhan Gargan, Seamus Hussey, Aideen Long, Patrick T. Walsh

**Affiliations:** ^1^National Children's Research Center, Children's Health Ireland (CHI) Crumlin, Dublin, Ireland; ^2^Trinity Translational Medicine Institute, Trinity College Dublin, Dublin, Ireland; ^3^Department of Paediatrics, Royal College of Surgeons of Ireland, Dublin, Ireland

**Keywords:** inflammatory bowel disease, Crohn's disease, ulcerative colitis, pediatric, T cell trafficking, infiltration

## Abstract

T cell subsets are considered central orchestrators of inflammation and homeostasis in the intestine and are established targets for the treatment of inflammatory bowel disease. While approaches aimed at the neutralization of T cell effector cytokines have provided significant benefits for pediatric and adult patients, more recent strategies aimed at inhibiting the infiltration of pathogenic T cell subsets have also emerged. In this review, we describe current knowledge surrounding the function of T cell subsets in pediatric inflammatory bowel disease and outline approaches aimed at targeting T cell trafficking to the intestine which may represent a new treatment option for pediatric inflammatory bowel disease.

## Introduction

Inflammatory Bowel Disease (IBD) is a heterogeneous autoimmune disorder characterized by chronic and relapsing inflammation of the gastrointestinal (GI) tract. The two main subtypes of IBD are Crohn's Disease (CD) and Ulcerative Colitis (UC) (1). Around 20% of IBD cases are diagnosed during childhood, mainly during adolescence (2). IBD disease phenotype and natural history differ depending on the age of onset (3). Pediatric (P)IBD can be subdivided into early onset (EO) IBD diagnosed after the age of 10, very early onset (VEO) IBD diagnosed under age 10 and infantile IBD diagnosed under the age of 1 (4). The global incidence of PIBD, primarily CD, is rising and CD is currently the most common form of PIBD (5, 6). Studies from countries including Ireland (7), Scotland (8), and Spain (9) have shown increased PIBD cases over time. The prevalence of PIBD is higher in western populations and highest in North America and Europe (10). The “hygiene hypothesis” suggests that lack of exposure to microbial infections in early life increases the risk of developing CD (11–13). Genetic susceptibility (with susceptibility loci in genes encoding mediators of the immune response to microbes such as *IL23R* and *NOD2*), environmental factors (such as childhood infections) and the gut microbiome all contribute toward the development of PIBD (14–16). Breast feeding can decrease the risk of PIBD (17) while exposure to antibiotics during pregnancy increases the risk of VEO-IBD (18), likely due to changes in the microbiota (19).

Compared with adult onset IBD, PIBD is associated with early progression rates. Pediatric UC is associated with more severe colitis at onset (20) and CD displays a more panenteric phenotype during adolescence, with upper GI symptoms more common (21). No curative treatment exists but current treatments [including exclusive enteral nutrition (EEN)] aim to improve the quality of life, mitigate the psychosocial effects and reduce the need for surgery (22, 23). Common IBD symptoms include diarrhea, abdominal pain and weight loss (2) but PIBD can lead to further complications such as height impairment, malnutrition, anemia and delayed puberty (24). Delayed treatment increases the risk of growth-related defects (25, 26) while prolonged treatment with immunosuppressive therapies increases the risk of opportunistic infection (27). In addition, population studies have revealed higher mortality rates in patients with pediatric onset IBD compared with the general population due to the occurrence of colorectal cancer and infections (28–32). This highlights the potential benefit of novel, more specific therapies for PIBD. Similar to the more extensively studied adult disease, PIBD is characterized by a dysregulated inflammatory response to bacteria in the gut and subsequent activation and infiltration of T cells. This review will discuss the role of T cells in the pathogenesis of PIBD, the mechanism of T cell trafficking to the gut and the opportunity to target T cell migration as a treatment for PIBD.

## CD4+ T Helper Cell Subsets in the Chronically Inflamed Intestine

In the healthy gut discrimination between pathogenic and commensal bacteria is achieved through interaction between epithelial and immune cells in the gut-associated lymphoid tissue (GALT) where specialized epithelial M (microfold) cells deliver antigens to underlying lymphoid follicles (LFs) (33). In the small intestine, LFs group together to form Peyer's Patches (PPs) which increase in number with age, peaking at puberty (34). Antigen presenting cells (APCs) such as Dendritic Cells (DCs) in PPs and LFs uptake antigens from the gut lumen and migrate to the mesenteric lymph nodes (MLN) where they initiate T cell responses (33). In the healthy gut, DCs preferentially promote regulatory T cell (Treg) responses which contribute to intestinal immune tolerance and homeostasis through the production of anti-inflammatory cytokines such as IL-10 and TGF-β (35).

In PIBD dysbiosis leads to pathogenic T helper type 1 (Th1), Th2, Th9, and Th17 cell responses and an imbalance of Treg cells. Forkhead box P3 (FOXP3)+ Treg cells play an essential role in intestinal tolerance and gut homeostasis (36). The importance of the FOXP3 transcription factor is highlighted by Immunodysregulation Polyendocrinopathy Enteropathy X-linked (IPEX) syndrome, a rare disease caused by mutations in the gene encoding FOXP3 which results in severe autoimmunity and intestinal disease at infancy and early fatality due to the loss of Treg cells (37–39). This phenotype has been attributed to a dysfunction in Treg subsets which are critical toward maintaining immune tolerance in the gut. Along these lines, decreased levels of FOXP3+ Treg cells have also been reported in peripheral blood from patients with mild, moderate and inactive PIBD (40). Separately, decreased levels of FOXP3+ Treg cells were found in the colonic Lamina Propria (LP) of pediatric CD patients compared to healthy controls, though these decreased levels were reversed upon treatment with the TNF-α inhibitor infliximab (41).

However, several studies have also reported contrasting results showing increased levels of FOXP3+ Treg cells in the intestinal LP of pediatric CD patients (42, 43). Increased FOXP3+ Treg cells were found to occur in association with elevated levels of TGF-β1, suggesting that they maintain their anti-inflammatory phenotype (42). Higher levels of FOXP3+ Treg cells were also found in the ileal mucosa of pediatric CD patients when compared with adult CD patients and significantly higher levels of FOXP3+ Treg cells were found in the ileum and colon of treatment naïve pediatric CD patients compared with healthy controls (44). In addition, higher levels of FOXP3+ Treg cells have also been demonstrated in the peripheral blood, as well as the inflamed intestinal mucosa of PIBD patients (45, 46). Interestingly, levels of FOXP3+ cells were reported to be reduced in the intestinal mucosa of PIBD patients in remission when compared to those with active disease, although the levels of circulating Tregs remained elevated (45). Overall, these studies suggest that intestinal levels of FOXP3+ cells are most elevated during active PIBD but are modulated upon treatment.

Although such elevated levels of Tregs might be expected to exert a more profound immunosuppressive effect in the inflamed PIBD intestine, it remains to be fully determined whether the function of these cells is intact, and if not, whether this may contribute to disease pathology. The precise and complex role of FOXP3+ Tregs in the context of PIBD remains to be fully uncovered. In particular, whether these cells can play an important role in restricting pathogenic inflammation and/or promoting homeostasis and resolution will be important. Of interest in this regard, and pointing to the importance of intact Treg function, is the observation that gut homing CD4+ Treg cells express high levels of retinoic acid inducible CD38 and the co-inhibitory receptor T-cell immunoglobulin and Immunoreceptor Tyrosine-based Inhibition Motif (ITIM) domain (TIGIT) (47). In PIBD, loss of TIGIT expressing CD38+ T cells in peripheral blood was found to correlate with shorter remission periods (47). Furthermore, other FOXP3-ve Treg subsets may also play significant roles. IL-27, which induces the differentiation of naïve CD4+ T cells into Type 1 regulatory T cells (Tr1) cells, is encoded in a susceptibility locus for EO IBD (48, 49).

It has been suggested that the loss of balance between anti-inflammatory FOXP3+ Treg cells and pro-inflammatory IL-17A expressing T cells (Th17 cells) can contribute to the pathogenesis of IBD (50). The pathogenic role of Th17 cells in gut homeostasis and inflammation is complex. Although originally considered pathogenic in nature, clinical trials evaluating specific strategies aimed at IL-17A neutralization in adult CD patients were found to result in a worsening of disease outcomes, at least in some patients (51). Notwithstanding these observations, a recent study showed an increase in Th17 cells in peripheral blood from patients with PIBD which was attributed to increased serum levels of the Th17-inducing pro-inflammatory cytokines IL-23 and IL-6 (40). Interestingly, one study showed higher serum levels of Th17 signature cytokines IL-17A and IL-22 in pediatric patients with UC compared to those with CD (43) while another study showed upregulation of IL-17A and IL-22 mRNA in the colon of patients with both UC and CD when compared with healthy controls (46). Furthermore, a recent study showed significantly higher serum levels of IL-17A in PIBD patients compared with controls (52). These studies suggest a pathogenic role for Th17 cells in PIBD.

In addition to more recent analyses of Treg and Th17 subsets, earlier studies focused on characterizing the influence of Th1 vs. Th2 responses in the pathogenesis of PIBD. Based upon observations in adult patients, CD has classically been characterized as being associated with elevated Th1 type responses, whereas UC is associated with an “atypical” Th2 type response (53, 54). While early reports indicated that a similar dichotomy could be observed in PIBD patients (55, 56), a more complex picture has since emerged. For example, the Th2 transcription factor GATA-3 and the Th1 signaling molecule signal transducer and activator of transcription (STAT)4 were found to be significantly upregulated in the mucosa of patients with UC, highlighting the possible importance of both Th1 and Th2 subsets in the pathogenesis of pediatric UC (57). In contrast, it has also been reported that levels of expression of IFN-γ were reduced in the peripheral blood of newly diagnosed pediatric CD patients (58). While such reports highlight a lack of clarity concerning the relative contribution of distinct T subsets toward the pathogenesis of PIBD, it is also noteworthy that the frequency at which Th subsets can be detected changes dramatically with age especially during childhood (59, 60).

While most data concerning the nature of Th responses in the context of PIBD have focused on the subsets described above, there is also mounting evidence that less well-characterized subsets may play important roles. For example, CD4+ effector T cell subsets expressing high levels of IL-9 (Th9 cells) have also emerged as potential important players in the pathogenesis of adult UC and in preclinical models of disease (61). However, whether this subset plays a significant role in the pathogenesis of pediatric UC remains to be determined.

## Other T Cell Subsets In The Chronically Inflamed Intestine

As well as the CD4+ T helper cell subsets described above, several other immune cell subsets have been reported to influence the balance between T cell tolerance and inflammation in the intestine. Group 3 Innate lymphoid cells (ILC3s) have been shown to specifically induce the destruction of activated commensal bacteria specific T cells (62). However, the expression of major histocompatibility complex (MHC) class II was found to be lower in ILC3 cells from patients with PIBD which could contribute toward the dysregulation of T cell responses and gut homeostasis (62). PIBD patients were also found to have fewer CD39 expressing intraepithelial CD8+ memory T cells and γδ T cells compared with non-IBD controls (63). CD39 degrades excessive extracellular ATP and helps maintain gut homeostasis (63). Pediatric patients with newly diagnosed UC but not CD were found to have higher circulating levels of activated CD4+ and CD8+ T lymphocytes expressing β1 integrin, correlating with biomarkers of mucosal and systemic inflammation (64). In addition, a further study demonstrated decreased Vδ2+ T cells in the peripheral blood of pediatric CD patients, but an increased infiltrate of integrin β7 expressing Vδ2+ T cells in the colon, which exacerbated inflammation through the release of TNF-α (65). Such studies highlight the potential therapeutic benefit of inhibiting pathogenic T cell trafficking to the gut.

The orchestrating role of different T cell subsets in mediating inflammation and homeostasis in the intestine underlines their potential as therapeutic targets in IBD. Further investigation into the specific roles of T cell subsets, beyond CD4+ T helper subsets, in UC and CD may optimize this therapeutic potential. In particular, efforts aimed at restricting the ability of pathogenic T cell subsets to traffic to and infiltrate intestinal tissues is an area of intense investigation.

## Mechanisms of T Cell Trafficking

Whether gut resident T cells play instructive roles in homeostasis or inflammation, they must first home specifically to the intestinal tissues. In order for lymphocytes to travel through the endothelium into a specific tissue, they undergo three main steps: rolling, adhesion and trans-migration or diapedesis. Rolling is mediated by selectins and supported by integrins, whereas integrins mainly facilitate firm adhesion and diapedesis. Chemokine signaling is also important to guide the cells toward the tissue and activate their adhesion molecules (66).

**Figure 1:**
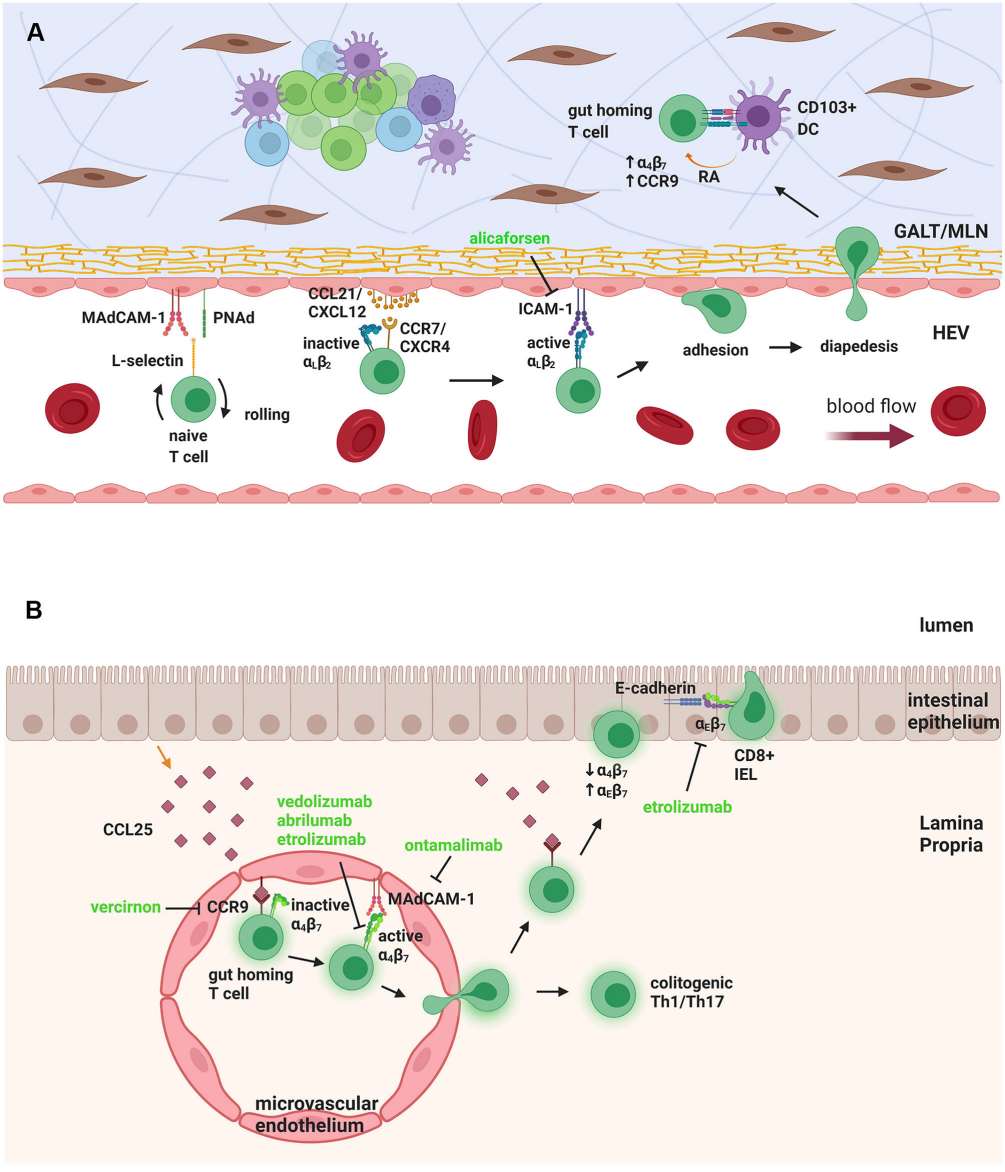
Naïve T-cell trafficking to GALT/MLN and gut homing T cell trafficking to LP and epithelium. **(A)** Naïve T-cells in the HEV need to undergo rolling, by interaction of L-selectin on their surface with MAdCAM-1 and PNAd on the endothelial cells. Binding of chemokines CCL21/CXCL12 to the chemokine receptors CCR7/CXCR4 activates αLβ2 integrin to bind to ICAM-1, leading to firm adhesion and diapedesis. When inside the GALT/MLN, the T-cell interacts with DCs that produce RA, to upregulate α_4_β_7_ and CCR9, giving it a “gut homing” phenotype. **(B)** The gut-homing T cell expresses CCR9, which binds to CCL25, produced by epithelial cells and anchored to microvascular endothelium. This causes the activation of α4β7 integrin, which binds to MAdCAM-1, leading to trans-migration of the T cell to intestinal LP. There it can stay, as a colitogenic Th1/Th17 cell, or move, again through CCR9-CCL25 interactions, toward the epithelium, where it downregulates α_4_β_7_ and upregulates α_E_β_7_, which binds to epithelial E-cadherin. The T cell then resides in the epithelial layer as a CD8+ IEL. Therapeutic targeting can be seen in green. GALT, Gut-associated lymphoid tissue; MLN, mesenteric lymph node; HEV, high endothelial venule; MAdCAM-1, mucosal addressin cell adhesion molecule-1; PNAd, peripheral node addressin; ICAM-1, intercellular adhesion molecule-1; DC, dendritic cell; RA, retinoic acid; IEL, intraepithelial lymphocyte. Figure created with Biorender.com.

For trafficking to the gut, initially, naïve T cells have to travel from the blood to GALT and MLN. GALT consists of the inductive sites of the intestinal tissue, mainly PPs and isolated lymphoid follicles (ILF) (67). Both GALT and MLN are associated with high endothelial venules (HEV) through which the T cells travel. HEV express mucosal addressin cell adhesion molecule-1 (MAdCAM-1) and peripheral node addressin (PNAd), which bind to L-selectin expressed on the T-cell surface (68–72) (Figure 1A). This allows tethering and rolling of the cells on the endothelial surface, which is then followed by firm adhesion, by binding of integrins α_4_β_7_ and lymphocyte function-associated antigen-1 (LFA-1; CD11a/CD18; α_L_β_2_) on the T-cell surface to their ligands on the endothelial cells, MAdCAM-1 and intercellular adhesion molecule-1 (ICAM-1), respectively (69). To promote this adhesion, integrins are activated by inside-out signaling *via* chemokine receptors, specifically CCL21 and CXCL12 binding chemokine receptors CCR7 and CXCR4, respectively, on T-cells. Firm adhesion is followed by trans-endothelial migration into the lymphoid tissue (69, 71, 73, 74).

The naïve T cells that enter the MLN and GALT become activated into colitogenic effector T cells, such as Th1 and Th17, but they also obtain a “gut homing” phenotype. This phenotype is characterized by upregulated adhesion molecules and chemokine receptors, especially α_4_β_7_ and CCR9, which bind to MAdCAM-1 and CCL25, respectively, on GALT and α_4_β_1_ and CXCR3, which bind to VCAM-1 and CXCL10 on activated endothelium (73–75). Upon entering GALT or MLN, lymphocytes encounter antigen through DCs, causing their polarization into effector cells and imprinting the gut homing phenotype (Figure 1A). A specific DC subset, which is CD103+, appears to be significant for this interaction and subsequent imprinting of the gut homing phenotype (76, 77). CD103+ DCs are derived from intestinal LP, and they express high levels of *Aldh1a2*, a gene encoding an isoform of retinaldehyde dehydrogenase (RALDH), which is mediator of the metabolic pathway converting vitamin A into retinoic acid (RA) (76–79).

Retinoic acid has been shown to be important for gut homing imprinting of both T and B lymphocytes, by upregulating α_4_β_7_ and CCR9 molecules (80, 81). Vitamin A deficiency results in a significant decrease in α_4_β_7_+ T cells in lymphoid organs and depletion of T cells from the small intestinal LP (80). Intestinal DCs and epithelial cells produce RA, which binds and signals through RA receptor-retinoid X receptor heterodimers expressed by recruited T and B cells (78, 79, 82). The RA receptor complex acts as transcription factor (78) contributing to the “gut homing” phenotype of GALT lymphocytes (80, 81). These B cells will then be activated into antibody producing plasma cells, which will undergo class switching into IgA producing cells in an RA dependent manner, and will reside in the intestinal mucosa (79, 81, 83). Gut tropism can be inhibited by LE540, a small molecule that blocks RA binding to RA receptor (81). FoxP3+ natural regulatory T cells (nTreg) can also be induced into a gut-homing phenotype in the MLN, further suggesting that in the steady state there is a balance of regulatory vs. effector T cells that might be disrupted in pathogenic conditions such as during IBD (84, 85). However, in adoptive transfer models of colitis, molecules such as L-selectin and CCR7, which allow homing to MLNs and GALT, seemed to be more important for Treg suppressive abilities than LP gut homing molecules, such as β_7_ integrin (86–88).

The gut-homing effector T cells re-enter the circulation and travel to the small intestine LP by binding to CCL25, mainly secreted by small intestine epithelial cells and anchored to the cell surface of LP microvascular endothelial cells. This in turn promotes activation of α_4_β_7_ for firm adhesion to MAdCAM-1 and migration to LP (69, 71–73). MAdCAM-1 is constitutively expressed by gut associated endothelium; however, its expression is upregulated in inflamed LP venules during both CD and UC (89). Some gut lymphocytes, mainly CD8+ T cells in the murine small intestine, reside inside the intestinal epithelial layer instead of the LP, and are termed intraepithelial lymphocytes (IEL). IEL need to further travel through the LP and the basal membrane to the intestinal epithelium, a process similarly mediated by CCR9 and CCL25 chemokine signaling (70, 90, 91). When IEL enter the epithelium, they downregulate α_4_β_7_ and upregulate α_E_β_7_, which binds to E-cadherin on intestinal epithelial cells, anchoring them to the epithelium (90) (Figure 1B). CCR9/CCL25 expression mediates rapid induction of α_E_β_7_ in murine CD8+ IEL and adhesion to E-cadherin (90).

Less is known on the mechanism of immune cell trafficking to the colon, compared to the small intestine. Activated CD8+ T cells required α_4_β_7_ to enter the colonic mucosa, but not CCR9, whereas CCL25 is expressed in very low levels in normal colon (92–94). On the other hand, blocking the CXCR4/CXCL12 chemokine signaling axis, inhibited lymphocyte adhesion to the colon as well as the small intestine (95). Moreover, vitamin A deficiency only reduced migration to the small intestine, not the colon, in a mesenteric lymphoblast transfer in rats, indicating RA is not required for “colon homing” (96). The orphan receptor GPR15 has been identified as a colon-homing receptor for human and murine effector CD4+ T cells, but not human Treg, making it a potentially attractive target for colitis intervention (97, 98). Expression of GPR15 is altered in UC, where it is enhanced in non-inflamed biopsies from UC patients when compared to inflamed biopsies (99). In contrast, CCL25 is upregulated in the inflamed colon of UC patients, and most infiltrating effector T cells (90%) in the inflamed tissue are CCR9+ compared to <10% in normal colon (100). This might indicate there is a shift in T cell chemokine receptor expression during active colitis, from GPR15 to CCR9 expression. In a mouse model of acute colitis, CCR9/CCL25 are upregulated in the colon, and play a regulatory role by controlling DC subsets balance (101).

Deficiency of β_7_ integrin in mice or antibody blockade of α_4_β_7_ or MAdCAM-1 molecules, severely impairs lymphocyte trafficking to GALT and intestine and ameliorates disease pathology in a mouse model of CD-like chronic ileitis (102–104). Although CCR9/CCL25 signaling is seemingly important for T cell trafficking to small intestinal LP, mice lacking any of these molecules have almost normal numbers of LP T cells, suggesting other mechanisms are in place to facilitate gut immune cell migration in the absence of this chemokine axis (105). Location appears to be an important determinant as to whether CCR9 plays a major role or not, as shown by competitive adoptive transfer experiments using both wild type and CCR9-/- α_4_β_7_+ T cells. These cells were more dependent on CCR9 for entry to the intestinal epithelium than the LP and in the proximal rather than the distal small intestine (94). Other chemokine receptor/chemokine pairs that are potentially important in recruiting T cells to the inflamed LP include CXCR3/CXCL10, CXCR4/CXCL12 and CCR6/CCL20 (95, 106). The expression of CXCR3 and its ligands CXCL9, CXCL10, and CXCL11 is upregulated in the inflamed colon of patients with PIBD (107). Blocking of CXCR4/CXCL12 axis reduced adherence of LP lymphocytes to intestinal microvessels, as shown by intravital microscopy in mice, in both normal conditions and during TNF-α induced inflammation, whereas blocking of CXCR6/CCL20 axis only affected adherence in inflamed tissue (95). CCL20 is upregulated in colon tissue of IBD patients and mice after DSS-colitis (108, 109).

## Strategies to Target T Cell Trafficking

Therapeutic approaches in PIBD are for the most part aimed at alleviating symptoms to facilitate healthier growth and development, while minimizing drug adverse effects, to improve quality of life for patients (110). Generally, the guidelines for the use of drugs in pediatric patients are based on adult studies, however, the pharmacokinetics and pharmacodynamics of drugs in children are different and doses have to be adjusted accordingly, for each child (111, 112). The therapeutic strategy for PIBD is to start gradually, with enteral nutrition intervention, and/or corticosteroid administration to achieve remission. However, in severe cases, more aggressive approaches are recommended, to induce mucosal healing and enter a state of “deep remission.” These approaches include immunomodulatory drugs, such as azathioprine and methotrexate (MTX), as well as biologics, mainly TNF-α inhibitors, and most commonly infliximab and adalimumab (113).

While the successful use of TNF- α inhibitors may impact T cell infiltration to the inflamed gut (114), there are currently two drugs in the clinic that specifically target immune cell trafficking approved for use in adult patients with IBD. These are both monoclonal antibodies designed to block integrins (Figure 1B). Natalizumab is a monoclonal antibody against α_4_ integrin subunit, and targets both α_4_β_7_ and α_4_β_1_ adhesion molecules. Although it can block lymphocyte trafficking to the gut, it also affects lymphocyte trafficking to the central nervous system, mediated by α_4_β_1_ integrin (115). As a result, natalizumab administration poses a risk of progressive multifocal leukoencephalopathy, caused by reactivation of the John Cunningham (JC) virus, for which 67.5% of adults with CD were seropositive, in range with the general population (116). On the other hand, vedolizumab poses no such risk, since it specifically targets the α_4_β_7_ integrin, with its effects on lymphocyte trafficking being limited to the gut (117). As such, vedolizumab demonstrates an excellent safety profile for use in IBD, with low rates of serious infections, malignancies and other adverse reactions (118). However, to date neither of these two agents have received regulatory approval for pediatric use, although they have been used off-label, and there have been limited reports on their successful use in PIBD (119, 120). Vedolizumab in particular, was more effective in pediatric UC than CD, and in anti-TNF treatment naïve patients, rather than those previously treated with TNF inhibitors (120). Currently, vedolizumab is mainly administered after failure of TNF-α antagonists, but these data strongly suggest it should be considered as a first line treatment for PIBD (121).

As well as the two approaches described above, a number of other strategies aimed at targeting T cell trafficking are also under clinical investigation (Figure 1; Table 1). Similar to vedolizumab, a second anti-integrin α_4_β_7_ blocking antibody, abrilumab, has undergone phase 2 trials in both UC and CD, with some positive outcomes (122, 123). Other small molecule antagonists of the α_4_ integrin subunit, AJM300, TRK-170 and Firategrast, have also undergone clinical study among adult IBD patient cohorts. A further approach currently under clinical investigation in IBD is etrolizumab (rhuMAb Beta7), a monoclonal antibody against the β_7_ integrin subunit, which blocks lymphocyte trafficking to the gut, mediated by α_4_β_7_ integrin, and retention of IEL on the enteric epithelium, mediated by α_E_β_7_ (124). Etrolizumab has shown very promising results in phase 1 and 2 clinical trials for UC (125, 126) and is currently on phase 3 clinical trials for both UC and CD. However, results for UC have been disappointing thus far. Significantly, etrolizumab is currently under investigation in phase 1 clinical trial in pediatric IBD patients, to assess pharmacokinetics, pharmacodynamics and safety (NCT03478956). Other molecules developed include an ICAM-1 adhesion molecule antisense blocking agent, alicaforsen (ISIS-2302) and an orally bioavailable CCR9 antagonist, vercirnon (GSK1605786; CCX282-B; Traficet-EN). Alicaforsen underwent phase 2 clinical trials in both UC and CD, with limited efficacy (127, 128), whereas vercirnon was in phase 3 clinical trials in CD, but failed to demonstrate efficacy (129, 130). An antibody targeting MAdCAM-1 is also being investigated, ontamalimab (PF- 00547659; SHP647), with promising phase 2 trials in UC, that is currently under phase 3 trials in both UC and CD (131). Finally, an oral sphingosine 1-phosphate receptor (S1PR) 1 and 5 agonist, ozanimod (RPC1063), has undergone phase 2 clinical trials in UC and CD and phase 3 trials are currently recruiting patients for moderate to severe UC and CD. Ozanimod is an S1PR agonist that can cause sequestration of lymphocytes into the secondary lymphoid organs and subsequent reduction of migrated lymphocytes in the gut and other organs, that is currently approved for treatment of multiple sclerosis (132).

**Table 1:** Drugs targeting lymphocyte trafficking currently under clinical trial.

**Drug**	**Target**	**Phase**	**Indication**	**Outcomes**
Abrilumab (AMG181)	Antibody against α_4_β_7_ integrin	2	UC and CD	Positive outcomes, particularly for UC
AJM300	Oral α_4_ integrin antagonist	3	UC	Phase 2 trial in UC positive
TRK-170	Oral α_4_ integrin antagonist	2	CD	Unknown
Firategrast (SB 683699)	Oral α_4_ integrin antagonist	2	CD	Unknown
Etrolizumab (rhuMAb Beta7)	Antibody against β_7_ integrin	3, 1 for PIBD	UC and CD, PIBD	UC results disappointing, some UC trials terminated
Alicaforsen (ISIS-2302)	ICAM-1 antisense inhibitor	2	UC and CD	Limited efficacy
Vercirnon (GSK1605786; CCX282-B; Traficet-EN)	Oral CCR9 antagonist	3	CD	Limited efficacy
Ontamalimab (PF- 00547659; SHP647)	Antibody to MAdCAM-1	3	UC and CD	Promising results in phase 2 trial in UC
Ozanimod (RPC1063)	Oral S1PR1 and 5 agonist	3	UC and CD	Preliminary results in UC positive

In conclusion, targeting of lymphocyte trafficking to the gut may represent a very promising novel therapeutic approach for PIBD, with fewer adverse effects than general immunomodulatory and anti-TNF therapies, due to its more selective nature. Targeting of adhesion molecules has been extensively studied in the clinic, with demonstrated efficacy among adult patients and further promising new agents currently in development. Other targets, such as chemokine receptors and S1PR, are also available, but further study is warranted to prove their efficacy and safety. While the overwhelming majority of these approaches have undergone clinical investigation among adult IBD patients, more extensive pediatric cohort trials are necessary before an evaluation of their appropriateness for use in PIBD.

## Author Contributions

All authors listed have made a substantial, direct and intellectual contribution to the work, and approved it for publication.

## Conflict of Interest

The authors declare that the research was conducted in the absence of any commercial or financial relationships that could be construed as a potential conflict of interest.
